# Do working parents in the United States expect work location to impact job and family satisfaction in the post-pandemic period? Evidence from a survey experiment

**DOI:** 10.3389/fsoc.2024.1368594

**Published:** 2024-03-20

**Authors:** Stephanie Moller, Leah Ruppanner, Jill E. Yavorsky

**Affiliations:** ^1^University of North Carolina at Charlotte, Charlotte, NC, United States; ^2^The University of Melbourne, Parkville, VIC, Australia

**Keywords:** remote work, work family conflict, job satisfaction, family satisfaction, survey experiment, vignette

## Abstract

The pandemic response allowed many parents in the United States and globally to work remotely for the first time ever which, for many, continued into the recovery. It is unclear whether, after a period when a large segment of the United States labor force worked remotely, remote work is viewed favorably or unfavorably among employed parents. We present results from a survey experiment assessing whether employed parents in the United States perceive that remote work will impact a hypothetical employed parents’ job and family satisfaction and, critically, whether perceptions of work–family conflict and anticipated job rewards mediate this relationship. We find that respondents who are also employed parents perceive that hypothetical employed parents who access remote work will report lower job satisfaction and higher family satisfaction. Perceptions of work–family conflict do not mediate this association. Rather, we find that job rewards (e.g., pay, promotion, etc.) fully mediate the relationship between remote work and perceived job satisfaction. Ultimately, this indicates that employed parents perceive that remote work will bring workers like them less pay and thus lower job satisfaction but greater family satisfaction. This extends arguments about remote work in the light of the conceptualization of a flexibility stigma and a flexibility paradox. Implications for practice and theory are discussed.

## 1 Introduction

The pandemic brought worldwide changes to people’s working situations, including increases in remote work and changes to their employment status via layoffs or resignations (Amanor-Boadu, 2022; Wigert and Agrawal, 2022). Indeed, the United States (U.S.) witnessed a historic number of workers quitting their jobs during the pandemic, with an average of 4 million resignations each month in 2021 (Society for Human Resource Management, 2022). Among those who remained employed, a large majority (70% at the height of the pandemic) of U.S. workers in telecommuting capable jobs worked from home (Wigert and Agrawal, 2022). Access to telecommuting was a critical resource to parents’, especially mothers’, continued employment during the pandemic (Collins et al., 2021). Yet, since pandemic restrictions have lifted in the U.S., many organizations have expanded requirements for in-person employment, though there is still a sizable uptick in remote employment, compared to the pre-pandemic period (Haan, 2023). Furthermore, many workers are returning to paid employment after the great resignation (Lee et al., 2023).

Amidst these changes, workers in the U.S. must decide whether to pursue remote or in-person employment, and organizations must decide whether to continue to offer remote work as a flexible workplace option. These types of challenges have plagued nations globally, and they are particularly salient in countries without protective policies for those who seek remote work. In countries like the U.S., there is a potential disconnect between employees’ and employers’ perceptions of remote work as employers are more likely to perceive that remote work undermines productivity because, unlike employees, employers do not consider commute time in calculations of productivity (Society for Human Resource Management, 2021). Given this documented disconnect between employers’ and employees’ evaluations of remote work, it is important for U.S. organizations to better understand workers’ perceptions of remote work as organizations must continue to hire and retain workers.

We examine whether working parents in the U.S. perceive different levels of work and family satisfaction in light of remote work because recent research suggests that workers believe that remote work can generate a workforce that is happier and more satisfied with work and personal life (Kantar, 2022; Kondratowicz et al., 2022). Satisfaction with one’s job is important because job satisfaction strengthens job commitment, enhances job performance, and lowers turnover (Brown and Peterson, 1993; Wright and Bonett, 2007). Satisfaction with one’s family is important because it increases overall well-being and results in more stable marriages (Mills et al., 1992; Burch, 2020). Yet, it is not clear whether and how working parents perceive that remote work would impact perceptions of job and family satisfaction, in part, because of the ways in which remote work is perceived to impact work–family conflict and job rewards.

Workers may perceive that remote work generates greater job and family satisfaction because it frees time to spend with loved ones; indeed, prior research illustrates higher satisfaction for remote workers (Kelliher and Anderson, 2010; Allen et al., 2015; Kim et al., 2020; Kantar, 2022; Qiu and Dauth, 2022). Yet, prior research has also established a flexibility paradox. According to this paradox, remote work has potential to facilitate working parents’ efforts to perform their dual roles of caregivers and paid workers; yet, it may simultaneously exacerbate exploitation of remote workers because the boundaries between work and home get blurred and work hours expand (Chung, 2022). As a result, working parents, particularly working mothers, may experience greater work–family conflict that may lead to lower satisfaction with work and family (Allen et al., 2000; Laß and Wooden, 2022). Remote work may also lower job satisfaction because it is associated with fewer job rewards. Indeed, remote work has historically been plagued by the flexibility stigma wherein remote workers are perceived as less committed to their jobs (Williams et al., 2013; Munsch et al., 2014; Chung, 2018). This results in fewer rewards which in turn lowers job satisfaction (Judge et al., 2010; Campisi, 2022).

Given the complicated outcomes of remote work in light of the flexibility paradox and the flexibility stigma and given the choices currently facing workers and employers, we conducted a study to assess perceptions of remote work among employed parents in the U.S. We present results from a survey experiment assessing whether employed parents in the U.S. perceive that remote work will impact a hypothetical employed parents’ job and family satisfaction and whether perceived work–family conflict and anticipated job rewards mediate this relationship. Specifically, we assess whether working parents expect remote work to lead to greater job and family satisfaction for a hypothetical working parent in a critical period—the pandemic recovery. Workers may perceive that remote work will lessen work–family conflict which translates into greater job and family satisfaction. Or, in alignment with the flexibility stigma, employed parents may perceive remote work as limiting job rewards—pay, promotions, and career progression—which may align with perceptions of lower job satisfaction. Or, in light of the documented flexibility paradox, employed parents may see remote work intensifying conflict between work and family because, while parents are able to take on domestic work during the workday, the demands of work increase and paid work hours expand. Thus, work and family demands simultaneously increase while work-family boundaries weaken. This could potentially lead employed parents to associate remote work with lower satisfaction. We test these relationships to contribute to our understanding of these theoretical frameworks (Chung, 2022), to better understand workers’ perceptions, and to offer practical insights for organizations during this period of adjustment.

In this novel experimental survey, a sample of employed parents in the U.S. (*n* = 518; heretofore referred to as respondents) are exposed to a vignette where a working parent’s employer re-opens offices post-pandemic. Our experimental design helps us understand the perceived negative consequences of working remotely for parents for a sample of respondents at an “arms-distance” (e.g., not themselves). This allows us to capture implicit biases in perceptions of remote work (Auspurg et al., 2017) for a group heavily impacted by these decisions—employed parents. In the vignette, the parent of young children decides whether they prefer to continue working in-person or remotely post-pandemic, and the boss permits the working parent to work at the preferred location. Our 2 × 2 design manipulates the gender of the working parent and the preferred work location (in-person or remote). After the vignette, we ask respondents to assess the hypothetical working parent’s family and job satisfaction, levels of work-to-family and family-to-work conflict, and perceptions of job rewards. This study allows us to determine whether respondents associate remote work with job and family satisfaction and whether work–family conflict and perceived job rewards help explain these associations.

Our experimental research allows us to uncover perceptions of remote work after this major exposure experience during the pandemic. It is imperative that organizations better understand how parents perceive remote work as perceptions inform behavior. Perceptions are not only developed out of direct experience, but they are also forged out of culturally derived biases. There is substantial literature that illustrates that remote work has historically been devalued because remote workers are perceived to be distracted during the workday as family responsibilities interfere with work responsibilities (Kraut and Grambsch, 1987; Munsch, 2016). These implicit biases shape how workers discuss remote work, how workers interact with colleagues who work from home, how employed parents approach the possibility of remote work, whether new and hopeful parents perceive that remote work is a potential long-term work strategy, and how parents of adult children mentor their children in terms of workplace strategies. Ultimately, our research strategy allows us to capture implicit biases in perceptions of remote work for employed parents (Auspurg et al., 2017) who are vulnerable to these biases and may be guiding these conversations. We do not ask respondents about their own experiences—whether remote work generates conflict and satisfaction in their own lives. This research is already feasible with standard survey methods (Schall, 2019), a methodology that is less effective at capturing implicit biases. Instead, we take an experimental approach and randomly assign respondents to a scenario where a working parent either works remotely or works in-person, and then we assess whether the respondent perceives that the worker faces conflict and is satisfied. Thus, we are building a deeper understanding of expectations of remote work on parents’ work experiences for the group most impacted—working parents.

## 2 Background

### 2.1 Remote work and satisfaction

There is substantial research documenting a flexibility stigma associated with remote work. Within this perspective, remote work is a status signal as it represents deviation from the ideal worker norm in the U.S., a norm that is singularly focused on paid work with few distractions, a norm that was forged within the male breadwinner model of employment (Blair-Loy, 2005; Williams et al., 2013). Remote work is associated with a flexibility stigma, in part, because it was historically perceived to be disproportionately pursued by parents, mainly mothers, to support continued employment after childbirth (Glass, 2004; Singley and Hynes, 2005). For working caregivers, remote work provides flexibility to manage competing work and family roles by placing workers in close physical proximity to family (Allen et al., 2013; Chung and Van Der Lippe, 2020). This allows workers the flexibility to multitask during work hours (Chung and Van Der Horst, 2018; Sherman, 2020) which is inherently distracting and in violation of the ideal worker norm. The flexibility stigma has generated a status marker that results in stereotyping, discrimination, and penalties, including negative performance evaluations, fewer promotions, and wage penalties (Cuddy et al., 2004; Glass, 2004; Munsch et al., 2014; Glass and Noonan, 2016; Munsch, 2016; Fuller and Hirsh, 2019; Emanuel and Harrington, 2021). For example, a UK study found that approximately one-third of workers believe that flexible work generates more work for others and results in fewer promotion opportunities (Chung and Van Der Lippe, 2020). As a result of this stigma, there has historically been a low uptake of remote work, and many remote workers were higher paid professional workers as opposed to workers who were searching for work-life balance (Williams et al., 2013; Chung and Van Der Lippe, 2020). Unsurprisingly, researchers have also found that this stigma is associated with lower job satisfaction (Cech and Blair-Loy, 2014). Thus, we predict that:^1^

*H*1: Study participants will perceive lower job satisfaction among the hypothetical employed parents who choose remote work (compared to the hypothetical employed parents who choose in-person work).

Chung (2022) extended the implications of the flexibility stigma in the conceptualization of a Flexibility Paradox. According to Chung (2022), the flexibility stigma is partially responsible for the paradoxical implications of remote work. Flexibility allows parents to remain in the labor market after childbirth, and it permits parents to commit more time to their work and family roles, but since the flexibility is stigmatized, workers overcompensate by working harder and many employers increase work demands. In fact, Chung (2022) argues that parents who work remotely spend more time on paid labor, while some parents, notably mothers, also spend more time on childcare. Thus, while working from home eases role transition, it also blurs role boundaries, potentially exacerbating work–family conflict. Thus, flexibility often expands work into family life. Thus, we predict that:

*H*2a: Study participants will perceive lower family satisfaction among the hypothetical employed parents who choose remote work (compared to the hypothetical employed parents who choose in-person).

Yet, we may not find support for this hypothesis derived from the flexibility paradox and may instead find that remote work is associated with higher family satisfaction because many workers think remote work will alleviate work and family conflict. Indeed, the stigma associated with flexible working arrangements stems from the assumption that remote work allows workers to better complete family-related tasks during the day, while they are supposed to be technically on the job, working. This might mean that people perceive that remote workers have fewer family-related tasks to be completed while they are spending time with their family (in the evenings after work, for example), leading to a happier home life. Thus, we propose a counter hypothesis:

*H*2b: Study participants will perceive higher family satisfaction among the hypothetical employed parents who choose remote work (compared to the hypothetical employed parents who choose in-person).

Remote work may be particularly crucial to mothers’ job and family satisfaction given that mothers are disproportionately responsible for domestic work, even when employed full-time (Hochschild, 2012; Möhring et al., 2021). As a consequence, remote work is historically considered a gendered work strategy to help mothers maintain employment as family demands intensify (Gornick and Meyers, 2003). The flexibility stigma is gendered with mothers experiencing greater discrimination from the stigma as they are perceived as less committed to their jobs and experience fewer promotions (Chung and Van Der Lippe, 2020; Villamor et al., 2023). Furthermore, during the pandemic, mothers who worked remotely increased the time spent caring for family (Chung, 2022). Thus, we expect our respondents to view remote work as a more critical resource for mothers’ job and family satisfaction than fathers’. We hypothesize:

*H*3: Perceptions of job (H3a) and family (H3b) satisfaction in light of remote work will be contingent on the hypothetical employed parent’s gender (as manipulated in the survey experiment).

### 2.2 Identifying the mechanisms

This research examines two mechanisms that might explain the relationship between remote work and satisfaction: work–family conflict and job rewards. In alignment with the Flexibility Paradox, employed parents may perceive that remote work would instigate work–family conflict because it has potential to create an environment where individuals are required to divide their resources, including time and attention, to competing demands (Allen et al., 2013) and remote work is associated with an increase in work hours (Chung, 2022). Since time resources are finite, conflict emerges when the roles compete. Indeed, both work and family place greedy, insatiable demands on time and attention (Chung, 2022). Consequently, many workers face competing devotions—to family and work—that make reconciling the two difficult (Blair-Loy, 2005). This is often referred to as border creep as the boundaries between work and family roles are blurred and ultimately are in conflict (Fonner and Roloff, 2010; Glavin and Schieman, 2012; Allen et al., 2013; Chung and Van Der Lippe, 2020). Thus, employed parents may have more critical perceptions of remote work and perceive that it will create work or family conflict. Yet, again, these relationships are complicated. While scholars have found a flexibility paradox, the flexibility stigma is based on the notion that remote work was historically perceived to facilitate role transition which can generate lower work–family conflict and reduce resource strain as commute time is repurposed to work or home demands (Glavin and Schieman, 2012; Allen et al., 2013; Chung and Van Der Lippe, 2020). This could ultimately enhance satisfaction with the job or family. Thus, while both the flexibility paradox and the flexibility stigma suggest a role for work–family conflict, they predict contradictory effects. Regardless, work–family conflict should serve as a mediator of remote work and job rewards. Thus, we hypothesize that:

*H*4a: Perceptions of work-family conflict will mediate the relationship between remote work and perceived job satisfaction for the hypothetical employed parents.

*H*4b: Perceptions of work-family conflict will mediate the relationship between remote work and perceived family satisfaction for the hypothetical employed parents.

To preview measurement, we include two measures of work–family conflict because some scholars are particularly interested in the domain in which conflict emerges. Work and family are unique domains that can either be the source or receiver of conflict. Work can interfere with family (known as work-to-family conflict, or WTF) or family can interfere with work (i.e., family-to-work conflict, or FTW). Madsen (2003) finds remote work lessens role strain which lowers both WTF and FTW conflict (Madsen, 2003). By contrast Laß and Wooden (2022) find remote work can expand work hours exacerbating WTF conflict (Laß and Wooden, 2022). Kim et al. (2020) also finds that remote work generates higher WTF conflict. In contrast, Allen et al. (2013) found that remote work reduces WTF conflict, but it increases FTW conflict. Scholars also argue that family roles are more permeable than work roles. Thus, WTF conflict is more common, in part, because work can more easily bleed into family life especially among professional workers whose work is enabled by technology (Ernst Kossek and Ozeki, 1998; Schieman et al., 2009). Yet, there is no research that assesses how people *perceive* remote work in terms of WTF or FTW conflict. It is also unclear how perceptions of the source of conflict align with perceptions of satisfaction. Some scholars argue that dissatisfaction occurs in the domain that causes the conflict (Amstad et al., 2011; Shockley and Singla, 2011; Hong et al., 2021), while others argue that dissatisfaction occurs in the domain receiving the conflict (Frone et al., 1992; Frone, 2003). Given the highly conflicting results of this literature, we do not develop *a priori* hypotheses regarding the domain from which conflict originates. However, we do operationalize work–family conflict with two measures that tap into the domains.

Finally, we juxtapose the mediating effects of work–family conflict on job satisfaction against the mediating effect of job rewards. According to the flexibility stigma, parents, especially mothers, who work remotely are often seen as less committed to their jobs and receive fewer job rewards, including promotions (Villamor et al., 2023). Higher rewarding jobs, including jobs with higher pay and opportunities for promotion, should be associated with greater job satisfaction (Beutell and Wittig-Berman, 1999; Thomas et al., 2004; Judge et al., 2010). The question remains as to whether employed parents have internalized the flexibility stigma and thus see working remotely as leading to fewer job rewards and thus lower job satisfaction for our hypothetical parent and whether the consequences spill into the family sphere.

From this, we derive our final hypotheses:

*H*5a: Perceptions of job rewards will mediate the relationship between remote work and perceived job satisfaction for the hypothetical employed parents.

*H*5b: Perceptions of job rewards will mediate the relationship between remote work and perceived family satisfaction for the hypothetical employed parents.

**Figure 1:**
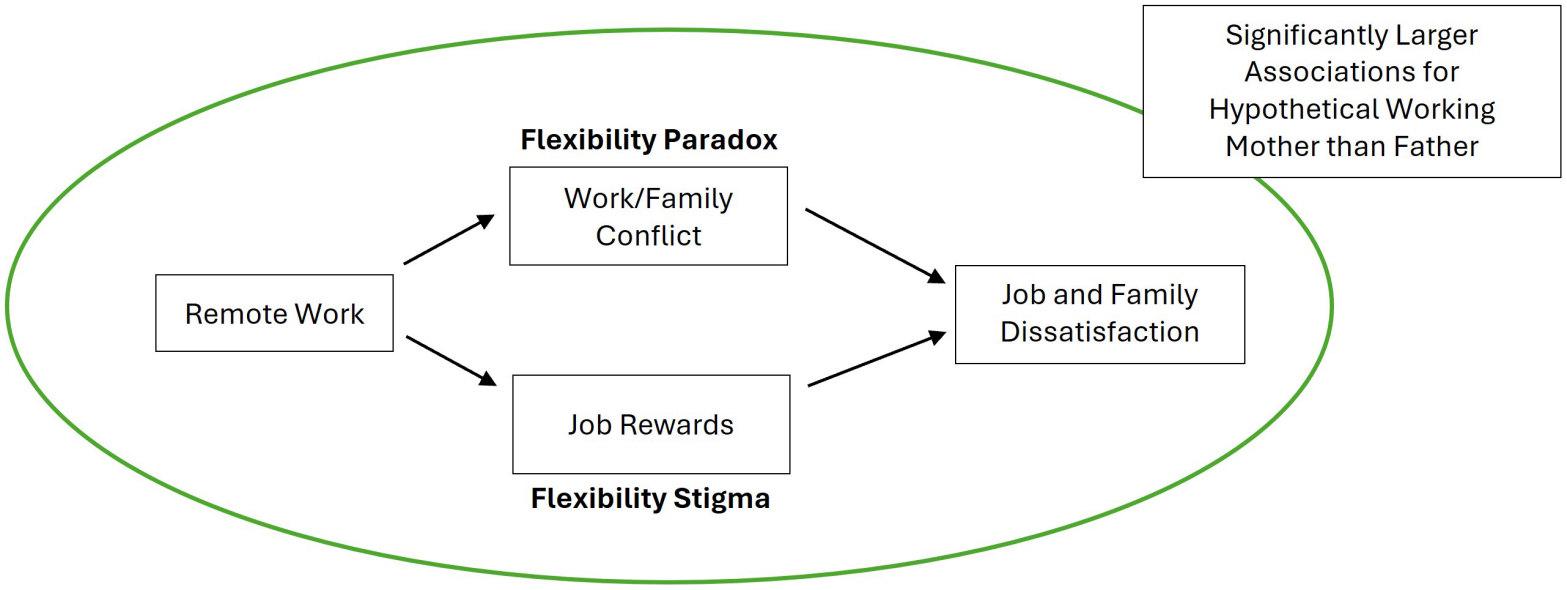
Graphical depictions of the flexibility paradox and flexibility stigma (H1, H2a, H3a, H4a, H4b, H5a, and H5b).

**Figure 2:**
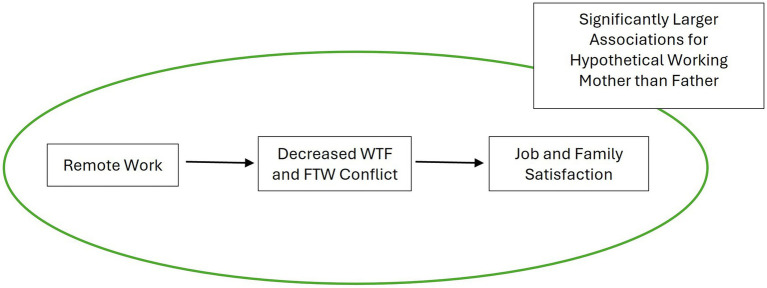
Graphical depictions of remote work benefits (H2b and H3).

To summarize, we have developed a series of hypotheses to explain the relationship between remote work and perceptions of work and family satisfaction (H1 and H2a; see Figures 1, 2 for a graphical depiction). We have also established that these effects should be gendered because the gendered nature of remote work has led to both the flexibility stigma and the flexibility paradox (H3). We expect remote work to be associated with lower work and family satisfaction because it is also associated with work–family conflict (H4a and H4b; per the flexibility paradox) and because it is associated with fewer job rewards (H5a and H5b; per the flexibility stigma). Yet, we also acknowledge that remote work is historically considered a workplace strategy that facilitates parents’ ability to care for family while working; indeed, this is the foundation of the emergence of the flexibility stigma. Thus, remote work could be associated with greater satisfaction (particularly in the family sphere) because it reduces work–family conflict (H2b).

## 3 Materials and methods

To test these hypotheses, we analyze data from a 2 × 2 factorial survey experiment, conducted in July 2022, where we manipulated the worker’s preferred work location status (work from home/work in-person at the corporate office) and worker gender (by assigning gender stereotypic names to the worker). Utilizing the Prolific online platform, we surveyed individuals who work full-time, ages 25–59, in the U.S. The sample corresponds with the working population on demographic characteristics, like gender (about half our sample is women, similar to the working population), though Whites are over-represented, and Hispanics are under-represented in our sample. Our sample is slightly younger than the working population and more highly educated, with 24% of people in our sample having an advanced degree (Flood et al., 2023; U.S. Bureau of Labor Statistics, 2023). As such, our sample aligns more closely with workers who typically have greater access to remote work, given that remote work is more common among white collar workers who tend to have higher education. Importantly, the sample of respondents were randomly assigned to experimental conditions.

The data were collected as part of a larger project examining the effects of status and power on workplace experiences. In the broader study, 1,201 participants were randomly assigned to one of 12 manipulations while ensuring a balanced sample between them. The study also involved manipulating the organizational authority of the target worker (high-level executive manager, mid-level manager, and no management responsibilities). To address the specific research question at hand, we collapse data across levels of this factor and assess the effects of gender and work preference regardless of organizational authority. All models control for management status. For the purpose of these research questions, we select working adults who have at least one child (*N* = 518). We narrowed the sample to working parents because we wanted to understand how those who are the most likely to use flexible work to accommodate family demands perceive this decision. Working parents may also be the biggest advocates for remote work, and thus, it is critical to identify the barriers they identify regarding parents’ use of this resource. This allows us to assess the extent that employed parents perceive conflict, rewards, and satisfaction for a hypothetical employee who works remotely versus in-person and who looks like them.

Using Qualtrics, we randomly assigned individuals to one of 12 manipulations with even representation between them. Respondents were presented with a version of the following scenario that holds age, number and age of children, marital status, occupation, work experience during COVID, and productivity constant. For ease of presentation, we present the scenario below with manipulated variables represented by one of the manipulated options in brackets. These manipulated options were presented randomly across respondents. [Michelle] could also be [John]. [Work from home] could also be [work in-person at the corporate office]. While we do not examine management status of Michelle and John in this manuscript, the vignette includes a manipulation of high-level executive manager, mid-level manager, and no management responsibilities. This manipulation is controlled in all statistical models.

[Michelle] is married, 35 years old, and has two young children, a toddler and an infant. [Michelle] works full-time in a demanding marketing job for a major U.S. corporation. [Her] role requires continuous interaction with her team. [Michelle] [does not have management responsibilities]. Typically, while [Michelle] is at work, their children go to daycare.

During the recent pandemic, [Michelle’s] company required individuals to work from home. This was helpful because [Michelle] and [her] spouse were unable to send their children to daycare during the pandemic, and [Michelle] was able to work at home alongside the young children. Nevertheless, [Michelle’s] productivity remained similar to her peers.

Because many of the pandemic-related restrictions have been lifted, Michelle’s two young children recently returned to full-time daycare. However, [Michelle] still has to deal with unexpected childcare responsibilities that occasionally arise during the workday. Michelle continues to work in the same demanding marketing position at the same company where [she] [does not have management responsibilities].

[Michelle’s] company is preparing to re-open the offices. Some employees are going to be required to return to the office, while others will be allowed to continue working from home. Each person will present their case on whether or not they should work at home or return to the office. The company will consider people’s preferences, alongside considerations for what is best for the success of the business.

[Michelle’s] preference is to [work from home].

Next, respondents were directed to assume that [Michelle’s] company granted [her] request to [work from home] in the upcoming year, and thus, [Michelle] was [working at home]. Respondents were then asked a series of questions to assess the outcome and mediation variables. We chose an occupation, marketing, that is mixed-gender in terms of who occupies the job in the US (U.S. Bureau of Labor Statistics, 2023). This choice helps reduce biases that may stem from participants thinking that an employed mother or father in the vignette was more or less suited to perform that work (based on skewed gender occupational compositions). We also chose this occupation because it is a common white-collar occupation, enabling our sample to have at least some knowledge about what this role might entail (rather than selecting a more obscure white-collar job). This role is similar to many other demanding white-collar jobs that require regular team interaction but a physical presence on-site is not absolutely essential to carrying out the work functions [unlike other jobs that *do* require employees to be on-site (e.g., a pharmacist or engineer who works on machinery)]. Thus, we strategically selected this job to minimize bias from occupational composition and standard work requirements.

Data were collected via Qualtrics utilizing the Prolific online platform. A recent study found that Prolific offers a subject pool that provides high quality data when considering comprehension, attention, and dishonesty (Eyal et al., 2021). To further ensure high quality data, we required all respondents to correctly answer questions assessing recollection of the manipulations. Each respondent was given two opportunities to recall all manipulations while accessing the vignette. Respondents who failed at least one of the manipulation checks twice (*n* = 6) were immediately removed from the study and did not respond to additional questions. All other individuals completed the entire study. To further ensure high quality data, we only included individuals in the final working sample who accurately answered at least one of two attention checks that assessed details in the vignette (the worker had young children and they were in formal daycare). Eight individuals from the original sample of 1,201 failed both attention checks or declined to answer the attention checks and were removed from the final analytic sample. We included three additional attention checks later in the survey. Respondents were removed from the sample if they failed to answer these checks or if they incorrectly answered two of them. This resulted in the loss of two additional respondents.

After viewing the scenario, respondents were asked to put themselves in the worker’s position and assess how satisfied the worker would be in the upcoming year with their job and with family life. *Job satisfaction and family satisfaction* were measured with two scales, both on a 5-point scale from completely dissatisfied to completely satisfied. Less than 5 percent of respondents perceive that workers would be dissatisfied with their job or with family, and once we divided this by work location status, the cells sizes become remarkably low, creating model instability. Therefore, we merge the bottom three categories for job satisfaction and family satisfaction, respectively. Substantively, this means our analyses compares whether the respondents perceive that the hypothetical working parents feel very or completely satisfied compared to those who do not feel this way (see Table 1 for descriptive statistics). This aligns with the literature as more satisfied individuals with job or family benefit on a wide variety of outcomes. As an example, according to GSS data, 56% of individuals who are very satisfied with their jobs are very unlikely to try to leave the job, compared to 31% of the moderately satisfied and 20% of the dissatisfied. GSS data also indicate that 40% of individuals who are very or completely satisfied with family are 2.5 times as likely as individuals who are only fairly satisfied to feel very happy with life in general (Davern et al., 2024).

**Table 1:** Descriptive statistics by remote work manipulation.

	Work from home		Work in-person			
Variable	Mean	Std. dev.	Mean	Std. dev.	Min	Max
Job satisfaction						
Neutral or dissatisfied	0.13	(0.34)	0.08	(0.27)	0	1
Very satisfied	0.69	(0.46)	0.73	(0.44)	0	1
Completely satisfied	0.18	(0.38)	0.19	(0.39)	0	1
Family satisfaction						
Neutral or dissatisfied	0.09	(0.29)	0.31	(0.46)	0	1
Very satisfied	0.68	(0.47)	0.57	(0.50)	0	1
Completely satisfied	0.23	(0.42)	0.12	(0.33)	0	1
Work-to-family conflict						
Hardly ever or never	0.18	(0.39)	0.11	(0.32)	0	1
Sometimes	0.63	(0.48)	0.58	(0.49)	0	1
Often or always	0.19	(0.39)	0.31	(0.46)	0	1
Family-to-work conflict						
Hardly ever or never	0.18	(0.39)	0.17	(0.38)	0	1
Sometimes	0.66	(0.48)	0.68	(0.47)	0	1
Often or always	0.16	(0.37)	0.15	(0.36)	0	1
Job rewards	11.64	(3.56)	14.48	(3.20)	5	20
Respondent’s gender						
Male/man	0.49	(0.50)	0.51	(0.50)	0	1
Female/woman	0.50	(0.50)	0.47	(0.50)	0	1
Other	0.00	(0.06)	0.01	(0.12)	0	1
Respondent’s age	40.60	(8.32)	40.64	(8.73)	25	59
Respondent’s race						
White	0.82	(0.39)	0.84	(0.37)	0	1
Black or African American	0.11	(0.31)	0.07	(0.26)	0	1
Asian	0.03	(0.18)	0.05	(0.23)	0	1
Other race	0.04	(0.19)	0.04	(0.19)	0	1
Respondent’s ethnicity						
Hispanic	0.04	(0.20)	0.08	(0.28)	0	1
Respondent’s education						
High school or less	0.07	(0.26)	0.06	(0.24)	0	1
Some college	0.18	(0.39)	0.15	(0.36)	0	1
Associate degree	0.11	(0.31)	0.11	(0.31)	0	1
Bachelor’s degree	0.42	(0.49)	0.41	(0.49)	0	1
Advanced degree	0.21	(0.41)	0.27	(0.45)	0	1
Has experience with remote work	0.68	(0.47)	0.76	(0.43)	0	1

*n* = 518.

Respondents were also asked, in the upcoming year, how often do you think the following would happen: Demands of the worker’s job would interfere with family life [*work-to-family (WTF) conflict*] and the demands of family life would interfere with the job [*family-to0work (FTW) conflict*]. Response options ranged from never to always (5-point scale). Given small cell sizes and model instability when we retain the 5 categories, the top two categories are condensed into a single category (often and always) and the bottom two categories are condensed into a single category (hardly ever and never).

We also asked about perceived *job rewards*. Our measure of job rewards is a sum of four items that assess how likely the boss would reward the worker with a (1) promotion, (2) salary increase, (3) high profile assignment, and (4) increased responsibilities. Categorical principal components analysis (CATPCA) was utilized to assess the internal consistency (alpha) of the scale. CATPCA relaxes the assumptions of linear relationships between variables, does not assume that variables are multivariate normal, and permits analyses of ordinal data (Linting et al., 2007). The job rewards scale has strong internal consistency with alpha above 0.9.

We analyzed satisfaction with partial proportional odds ordinal logistic regression. The traditional ordered logit model assumes parallel lines across levels of the dependent variable. The partial proportional odds model allows individual coefficients for a single independent variable to vary across levels of the dependent variable. We run the proportional odds model with GLOGIT2 in STATA (Williams, 2006). To test the proportional odds assumption for each variable, we use the autofit option with *p* < 0.025. This follows the advice of Williams (2016) as the cutoff of *p* < 0.05 is not sufficiently stringent when testing multiple effects. The proportional odds assumption is not violated in all models. When the proportional odds assumption is not violated, we revert to the standard ordered logit model. The tables include notes that clarify whether the proportional odds assumption is violated for each model. All models control for *gender* of the respondent (man, woman, and non-binary), *race* (white, black, Asian, and other race), *ethnicity* (Hispanic and non-Hispanic), *age*, *prior experience working remotely* (yes and no), and *highest education* (some college or trade school, associates, Bachelor’s degree, and advanced degree).

## 4 Results

### 4.1 Job and family satisfaction: remote work by gender of hypothetical parent

To test whether remote work is associated with work and family satisfaction, we present results from the partial proportional odds ordered logistic regressions. The first set of results, presented in Table 2, test the first three hypotheses by modeling the effects of remote work and gender. Models 1–2 present the results for job satisfaction; Models 3–4 present the results for family satisfaction. Models 1 and 2 do not violate the proportional odds assumption and default to a standard ordered logit model. The proportional odds assumption is violated for control variables in Models 3 and 4. Our first hypothesis (H1) predicts that study participants who viewed the vignette will expect lower levels of job satisfaction for the hypothetical parent who works from home, compared to in-person. Model 1 confirms this hypothesis demonstrating a negative effect of work from home for job satisfaction. Looking at the odds ratio, respondents were 35% [(1–0.65)*100] less likely to rate a hypothetical parent who worked from home higher on job satisfaction compared to respondents who rated a hypothetical working parent who worked in person. This is supportive of the expectations of the flexibility stigma as remote work is stigmatized and thus associated with lower job satisfaction. We then hypothesized that the gender of our hypothetical working parent would structure this relationship, but Model 2 documents a non-significant interaction term meaning our respondents do not expect a working mother to benefit more from remote work in terms of job satisfaction (H3 not supported). Models 3 and 4 test these relationships for family satisfaction. We find that employed parents expect the hypothetical parent to benefit from remote work in terms of family satisfaction (H2a rejected and H2b confirmed). In fact, when the hypothetical working parent works from home, they are three times as likely to be in higher levels of family satisfaction. These results align with the flexibility stigma which is based on the assumption that remote work allows working parents to care for family demands while working. While the flexibility stigma is mostly concerned with lower productivity and job commitment among remote workers, the stigma rests on the assumption that remote work fosters the simultaneous fulfillment of work and family roles that are conducive to work life balance. These results also suggest that respondents do not perceive of the paradox, wherein remote work allows paid work to bleed into family time. In contrast to both the flexibility paradox and flexibility stigma, we do not find that respondents expect our hypothetical working mother to benefit more than our hypothetical working father (H3 rejected). In separate analyses (not shown), we assessed whether the gender of the survey respondent interacted with the hypothetical worker’s gender and did not find a significant effect for either measure of satisfaction.

**Table 2:** Predicted coefficients, robust standard errors, and odds ratios for job and family satisfaction from partial proportional odds ordered logistic regression with robust standard errors, with gender interaction.

	Job satisfaction		Family satisfaction	
		Model 1				Model 2				Model 3				Model 4			
		Coef	*SE*		OR	Coef	*SE*		OR	Coef	*SE*		OR	Coef	*SE*		OR
Work location status																
Work from home	−0.42	(0.21)	^*^	*0.65*	−0.42	(0.28)		*0.66*	1.12	(0.20)	^***^	*3.00*	1.30	(0.26)	^***^	*3.67*
Gender																
Michelle	0.11	(0.20)		*1.3*	0.12	(0.26)		*1.12*	0.27	(0.19)		*1.35*	0.45	(0.27)		*1.58*
Interaction																
Work from home * Michelle	–	–		–	0.00	(0.41)		*0.99*	–	–		–	−0.38	(0.38)		*0.68*
AIC	800				802				890				891			

^***^p < 0.001, ^**^p < 0.01, ^*^p < 0.05 based on coefficient *z*-tests; both models control for respondents’ gender, age, race, ethnicity, work from home experience, and education, along with manipulated gender and management status; proportional odds assumption is not violated for Models 1–2, and these models revert to the standard ordered logit model with robust standard errors; proportional odds violated for the control variables, manipulated manager and respondents’ age and race in Models 3–4; *n* = 518. The italicized values under the OR column are odds ratios.

### 4.2 Job and family satisfaction: the mediating effects of work–family conflict, family–work conflict and job rewards

Table 3 presents the partial proportional odds ordered logistic regressions results to assess the mediating effects of work–family conflict, divided into work-to-family (WTF) conflict and family-to-work (FTW) conflict as well as job rewards. Model 1 presents the total effect of work location status (comparing remote work to in-person work) from Model 1 in Table 2. Model 2 then examines the effects of the measures of work–family conflict. Model 3 tests the measure of job rewards. Model 4 includes the combined model. The proportional odds assumption is not violated in Models 1 and 3; these models revert to the ordered logit model. The proportional odds assumption is violated for the sometimes category of WTF conflict in Models 2 and 4.

**Table 3:** Predicted job satisfaction from partial proportional odds ordered logistic regression with robust standard errors.

	Model 1				Model 2				Model 3				Model 4			
	Coef	*SE*		OR	Coef	*SE*		OR	Coef	*SE*		OR	Coef	*SE*		*OR*
Work location status																
Work from home	−0.42	(0.21)	^*^	*0.65*	−0.55	(0.22)	^*^	*0.58*	0.18	(0.21)		*1.19*	0.06	(0.24)		*1.07*
Gender																
Michelle	0.11	(0.20)		1.12	0.10	(0.21)		*1.10*	0.08	(0.21)		*0.22*	0.06	(0.22)		*1.06*
Work-to-family (WTF) conflict							^								^	
Sometimes (outcome: 1 vs. 2 and 3)	–	–		–	0.07	(0.44)		*1.07*	–	–		–	0.08	(0.48)		*1.08*
Sometimes (outcome: 1 and 2 vs. 3)	–	–		–	−1.27	(0.37)	^**^	*0.28*	–	–		–	−1.21	(0.43)	^**^	*0.30*
Often/always	–	–		–	−0.76	(0.41)		*0.47*	–	–		–	−0.71	(0.45)		*0.49*
Family-to-work (FTW) conflict							^								^	
Sometimes	–	–		–	−0.52	0.33		0.60	–	–		–	−0.52	(0.36)		*0.59*
Often/always	–	–		–	−1.40	0.46	^***^	0.25	–	–		–	−1.40	(0.48)	^**^	*0.25*
Job rewards	–	–		–	–	–		–	0.24	(0.04)	^***^	*1.27*	0.23	(0.04)	^***^	*1.26*
AIC	800				759				746				710			

^***^p < 0.001, ^**^p < 0.01, ^*^p < 0.05, based on coefficient *z*-tests; ^*p* < 0.05 based on chi-square test of the overall effect; all models control for respondents’ gender, age, race, ethnicity, work from home experience, and education, along with manipulated management status; outcome 1 is neutral or dissatisfied, outcome 2 is very satisfied, outcome 3 is completely satisfied; proportional odds assumption is not violated for Models 1 and 3, and these models revert to the standard ordered logit model with robust standard errors; proportional odds assumption is violated for the sometimes category of work-to-family conflict in Models 2 and 4; *n* = 518.

We hypothesized that WTF and FTW conflict would mediate the relationship between remote work and job satisfaction (H4a). We find that both WTF and FTW conflict predict lower satisfaction. To clarify the interpretation of the results for the sometimes category of WTF conflict, there are two sets of parameters because the proportional odds assumption is violated. When respondents perceive that the hypothetical worker sometimes experiences WTF conflict, they are not significantly less likely (given the non-significant coefficient) to also perceive that they hypothetical worker will be in the top two categories of satisfaction (very and completely satisfied), but they are significantly less likely (coef = −1.27) to be in the top category (completely satisfied), compared to the other categories of satisfaction. Thus, when respondents perceive that the hypothetical workers sometimes experiences WTF conflict, compared to rarely or never, they are significantly less likely to rate the hypothetical workers as completely satisfied with their jobs. The non-significant effect for the often/always category of WTF conflict (coef = −0.76) suggests that when the respondent perceives that the hypothetical working parent regularly experiences WTF conflict, there is not a significant association with job satisfaction. The results differ for FTW conflict. When the respondent perceives that the hypothetical working parent sometimes experiences FTW conflict, there is not a significant association with job satisfaction, but when the respondents perceive regular FTW conflict, there is a perception of significantly lower job satisfaction (coef = −1.40). Yet despite the significant effects of WTF and FTW conflict, neither of these variables mediate the relationship between work from home status and job satisfaction. In fact, once measures of work–family conflict are added in Model 2, the effect of work location becomes stronger in the negative direction. In separate analyses (not shown) we assess the effects of WTF and FTW conflict in separate models and find that the coefficient for remote work is stable when FTW conflict is included, and it becomes more strongly negative when WTF conflict is included. Thus, we do not find support for hypothesis 4a. In contrast to expectations, measures of perceptions of work–family conflict do not mediate the relationship between remote work and perceptions of job satisfaction for employed parents.

Hypothesis 5a predicts that job rewards will mediate the relationship between remote work and perceived job satisfaction, per expectations from the flexibility stigma. Model 3 illustrates that the measure of job rewards is significant. When respondents perceive that the hypothetical working parents receives greater job rewards, they also perceive greater job satisfaction (odds ratio = 1.27). Furthermore, work location status becomes non-significant when perceived job rewards is included in the model (support for H5a) and this is robust net of the inclusion of perceived work–family conflict (see Model 4). This suggests that remote workers are perceived to be less satisfied with their jobs because they are expected to receive fewer job rewards, in alignment with the flexibility stigma.

Table 4 presents the results for family satisfaction. Model 1 presents the total positive effect of work location status (comparing remote work to in-person work) on family satisfaction (a replicate of Model 3 in Table 2). Model 2 adds measures of work–family conflict. Model 3 tests job rewards, and Model 4 is the combined model. The proportional odds assumption is violated for at least one variable in all models. We see in Model 2 that perceived WTF conflict is associated with lower family satisfaction. According to the odds ratio, when respondents perceive that the hypothetical working parent sometimes experience work-to-family conflict, they also expect the hypothetical working parent to be 24% [(1–1.24)*100] less likely to be highly satisfied with family, compared to respondents who perceive that the hypothetical working parent never or rarely experiences WTF conflict. When they perceive that the hypothetical working parents often or always experiences work-to-family conflict, they expect the parent to be substantially less likely to be very or completely satisfied with their jobs (coef = −2.70) and also less likely to be completely satisfied (coef = −0.98). The overall effect of all categories of perceived FTW conflict does not significantly predict family satisfaction. Thus, we find that WTF but not FTW conflict predicts family satisfaction. Once measures of work–family conflict are controlled, the association between work location and family satisfaction remains strong (as the odds ratio is stable, changing minimally from 3.0 to 2.98). Thus, consistent with the results for job satisfaction (Table 3), we do not find that measures of work–family conflict mediate the relationship between remote work and family satisfaction (failing to support H4b).

**Table 4:** Predicted family satisfaction from partial proportional odds ordered logistic regression with robust standard errors.

	Model 1				Model 2				Model 3				Model 4			
	Coef	SE		*OR*	Coef	SE		*OR*	Coef	SE		*OR*	Coef	SE		*OR*
Work location status																
Work from home	1.12	(0.17)	^***^	*3.00*	1.09	(0.21)	^***^	*2.98*	1.52	(0.21)	^***^	*4.56*	–	–		–
Work from home (outcome: 1 vs. 2 and 3)	–	–		–	–	–		–	–	–		–	1.95	(0.23)	^***^	*7.05*
Work from home (Outcome: 1 and 2 vs. 3)	–	–		–	–	–		–	–	–		–	1.03	(0.28)	^***^	*2.81*
Gender																
Michelle	0.27	(0.19)		*1.30*	0.34	(0.20)		*1.40*	0.24	(0.19)		*1.26*	0.29	(0.20)		*1.34*
Work-to-family (WTF) conflict							^								^	
Sometimes	–	–		–	−1.24	(0.35)	^***^	*0.29*	–	–		–	−1.19	(0.37)	^**^	*0.30*
Often/always (outcome: 1 vs. 2 and 3)	–	–		–	−2.70	(0.42)	^***^	*0.07*	–	–		–	−2.64	(0.44)	^***^	*0.07*
Often/always (outcome: 1 and 2 vs. 3)	–	–		–	−0.98	(0.42)	^*^	*0.38*	–	–		–	−1.03	(0.43)	^*^	*0.36*
Family-to-work (FTW) conflict																
Sometimes	–	–		–	−0.35	(0.31)		0.70	–	–		–	−0.32	(0.32)		*0.73*
Often/always	–	–		–	−0.84	(0.41)	^*^	0.43	–	–		–	−0.80	(0.42)		*0.45*
Job rewards	–	–		–	–	–		–	0.13	(0.03)	^***^	*1.14*	0.13	(0.03)	^***^	*1.14*
AIC	890				820				871				804			

Outcome 1 is neutral or dissatisfied, outcome 2 is very satisfied, outcome 3 is completely satisfied; ^***^p < 0.001, ^**^p < 0.01, ^*^p < 0.05, based on coefficient *z*-tests; ^*p* < 0.05 based on chi-square test of the overall effect; all models control for respondents’ gender, age, race, ethnicity, work from home experience, and education, along with manipulated management status; proportional odds assumption violated for the often/always category of work-to-family conflict in Models 2 and 4 and for work from home in Model 4; proportional odds violated for the control variables: respondent’s race, respondent’s age and manipulated manager status in models 1 through 3; proportional odds also violated for the control variable: respondent’s ethnicity in model 2; *n* = 158. The italicized values under the OR column are odds ratios.

We expect job rewards to mediate the relationship between remote work and family satisfaction (H5b) but our results from Models 3 and 4 in Table 4 counter this hypothesis. Our respondents expect employed parents who receive more job rewards to report greater family satisfaction but the positive association between remote work and family satisfaction remains robust, significant and even strengthens. Thus, unlike the results for job satisfaction, job rewards do not mediate the relationship between remote work and family satisfaction (H5b rejected for family satisfaction). Interestingly, according to the odds ratio, once job rewards are controlled (in Model 4), respondents perceive that the hypothetical working parents who works from home is 4.56 times as likely as the hypothetical working parent who works in person to be at a higher level of job satisfaction. Thus, respondents expect working from home to bring greater family satisfaction net of anticipated job rewards. Once we include all variables in the final model, we see that working from home is robustly associated with greater family satisfaction, perceptions of work-to-family conflict is associated with lower family satisfaction, perceptions of family-to-work conflict is not related to family satisfaction; and greater job rewards is associated with greater family satisfaction. It is important to note that the proportional odds assumption is violated for work from home in Model 4. Despite this, work from home is significantly related to family satisfaction. The relationship is strongest when comparing very and completely satisfied to other categories, than when comparing completely satisfied to all other categories. More specifically, the odds ratio indicates that respondents perceive that working parents who work from home are 7.05 times as likely to be very or completely satisfied with family compared to working parents who work in person. Furthermore, respondents perceive that working parents who work from home are 2.81 times as likely to be completely satisfied with family compared to working parents who work in person.

## 5 Discussion and conclusion

This study set out to assess whether employed parents in the U.S. perceive that remote work fosters greater satisfaction with job and family in the post-pandemic period, whether work–family conflict and job rewards mediate this relationship and whether fathers are expected to experience these relationships differently than mothers. This research overcomes some of the challenges faced by survey research by presenting working aged adults with a scenario where a worker chooses to either work from home or work in-person at the corporate office. The respondent must then decide the extent that the worker would face work–family conflict and the degree of job and family satisfaction. We are not asking respondents about their own experiences but rather the realities they expect a working parent to face if allowed to work remotely. We select working parents to understand how they perceive these experiences to better contextualize the benefits and barriers parents themselves perceive about remote work. This provides an understanding of the constraints working parents perceive for other working parents to capture implicit bias. We find that our respondents perceive that remote workers are less likely to be very satisfied with their jobs and are more likely to be very satisfied with family, compared to in-person workers; but contrary to expectations, measures of work–family conflict do not mediate the relationship between remote work and satisfaction with job or family. Rather, we find employed parents expect remote work to bring fewer job rewards and, as a consequence, lower job satisfaction. They expect a career penalty in terms of fewer rewards for working from home and this penalty to be equivalent for working mothers *and* fathers. Yet, they also expect parents working remotely to experience significantly higher family satisfaction net of any changes to work–family conflict and impacts on job satisfaction.

This speaks directly to conceptualizations of the flexibility stigma and the flexibility paradox. The flexibility stigma suggests that remote work is stigmatized because remote workers spend more time on family responsibilities. This ability to combine work and family roles could potentially generate greater work life balance and yield higher family satisfaction. On the flip side, as a result of this stigma, remote workers could be expected to receive fewer rewards which could generate lower job satisfaction. In support of this perspective, we find that employed parents expect remote work to bring parents fewer job rewards and less satisfaction with the job, but they also have greater family satisfaction despite fewer rewards. These findings support the flexibility stigma but also indicate that these relationships are complex based on the domain of satisfaction (e.g., work vs. family).

The research on the flexibility paradox was developed as an expansion of research on the flexibility stigma. In essence, the flexibility stigma encourages remote workers to both self-exploit and accept increased exploitation from the employer as workers attempt to counter the stigma. Thus, finding support for the flexibility stigma does not necessarily contradict the flexibility paradox. However, the flexibility paradox further centralizes the role of work–family conflict. If our respondents had centralized the perception of greater work–family conflict in their assessments of the hypothetical workers then work–family conflict would have served as a robust mediator, an effect that is not supported by our models. Thus, when conducting research on workers’ perceptions, we find that the flexibility stigma is top of mind, but working parents are not clearly aware of the flexibility paradox found in recent research. Importantly, in the vignette, we primed respondents to see the potential for work–family conflict as the vignette notes that the parent “still has to deal with unexpected childcare responsibilities that occasionally arise during the workday.” Even with this prompting, work–family conflict is not a robust mediator. Furthermore, we do not find gender matters in structuring our respondents’ responses. Rather, study participants, who are parents, identify the same opportunities and constraints to parents’ working remotely regardless of gender. It may be that their direct experience with remote work may structure these responses—mothers and fathers who work remotely themselves may see the work-family challenges associated with this decision. We do not measure their own experiences, a limitation of this study and a direction for future research.

Some theorists are particularly interested in how the source of work–family conflict impacts satisfaction among workers; some theorists argue that lower satisfaction is found in the domain that causes the conflict (i.e., the source domain perspective). Other theorists suggest that lower satisfaction is found in the domain receiving the conflict (i.e., the cross-domain perspective). Our research does not test these theories directly as we do not assess workers’ experiences through traditional survey methods. However, this experimental design, focused on perceptions, allows us to extend these theories to *perceptions* of satisfaction in light of work–family conflict. We find more robust support for a cross-domain theoretical model as perceived family-to-work conflict is a robust predictor of lower perceived job satisfaction and perceived work-to-family conflict is a robust predictor of perceived family satisfaction.

This study is not without limitations. The movement away from remote work among major organizations in the U.S. continues into the pandemic recovery meaning remote work is increasingly less accessible over time. Thus, our respondents may view a parent who chooses to work remotely as even more vulnerable to fewer job rewards and worse job satisfaction as a consequence. Further, we restrict our sample to employed parents given their shared lived experience. Employees who are not parents may view these experiences differently. Thus, additional subgroup analyses are warranted. We also do not include hybrid options for remote work (e.g., swing shifts, part-day, set office days) which may also structure how our respondents view the rewards, conflict and satisfaction associated with working from home. Finally, we selected a job—marketing—because of its gender balance. Jobs in other industries, especially those with higher concentrations of remote work may lead our study participants to view greater satisfaction with and fewer penalties for our hypothetical working parent. By contrast, those with a higher concentration of men may result in harsher penalties. These limitations point to clear directions for future research.

Ultimately, our results are clear—we found that employed parents in the U.S. perceive that when a parent chooses to work from home, they will be penalized by their bosses in terms of career rewards, ultimately leading to lower job satisfaction. They also expect remote work to bring greater family satisfaction. Given that many workers prefer to continue to work remotely post-pandemic, remote work is associated with greater family satisfaction, and many organizations have invested heavily in infrastructure to permit remote work, it is imperative that organizations think about ways to ensure that remote work is equally rewarded and considered satisfactory employment. One strategy is for companies to collect employment-related (pay, promotion, etc.) data on remote and non-remote workers and then share data with employees in the company, including efforts to correct any discrepancies if found to exist. This could help workers have a more informed perspective of whether they could be penalized for working remotely. Moreover, our work suggests that it is critical for organizations to think carefully about how to roll out remote work options to their employees. Many organizations have remote work as an opt-in program. Instead, companies could structure the program to be the default option and employees could opt-out if they would like. Designing the program to be opt-out, instead of opt-in, might reduce stigma around working remotely—and may help to reduce workplace penalties too if the program is thought of as the norm work arrangement, rather than a special privilege granted to employees who are supposedly not “ideal workers.” Although our sample draws upon US parents, our findings may replicate in countries that are similar to the US, or those with legacies of limited access to remote work and long work hours. Additional cross-national research would illuminate the replicability of these findings in countries with different approaches to remote work access.

Finally, we do not find gendered effects for our hypothetical employees. The impact of remote work on perceptions of work and family satisfaction is comparable for mothers and fathers. Yet, we know that in the labor force, women remain more likely to request remote work arrangements than fathers given that they continue to bear a disproportionate share of household responsibilities. Thus, while the effects are the same, women remain more likely to experience the effects of remote work which makes them vulnerable. At the same time, fathers are increasingly accessing remote work, which may penalize them and discourage its use. Ultimately, equally valuing working parents including those who work remotely is tantamount.

## Data availability statement

The datasets presented in this article are not readily available. According to the IRB protocol, only research cleared by their IRB can access the de-identified data. Requests to access the datasets should be directed to stephanie.moller@charlotte.edu.

## Ethics statement

The studies involving humans were approved by the UNC Charlotte Institutional Review Board. The studies were conducted in accordance with the local legislation and institutional requirements. The participants provided their written informed consent to participate in this study.

## Author contributions

SM: Writing – review & editing, Writing – original draft. LR: Writing – review & editing, Writing – original draft. JY: Writing – review & editing, Writing – original draft.

## Footnotes

^1^Note, all of our hypotheses refer to employed parents *of small children*, as we describe in greater detail in the materials and methods section.
